# Physical exosome:exosome interactions

**DOI:** 10.1111/jcmm.13479

**Published:** 2018-01-29

**Authors:** Elie Beit‐Yannai, Saray Tabak, W. Daniel Stamer

**Affiliations:** ^1^ Clinical Biochemistry and Pharmacology department Ben‐Gurion University of the Negev Beer‐Sheva Israel; ^2^ Duke Eye Center Duke University Durham NC USA

**Keywords:** exosome, zeta potential, hypothesis, signalling, physical interaction

## Abstract

Exosomes are extracellular nanovesicles that mediate a number of cellular processes, including intracellular signalling. There are many published examples of exosome–exosome dimers; however, their relevance has not been explored. Here, we propose that cells release exosomes to physically interact with incoming exosomes, forming dimers that we hypothesize attenuate incoming exosome‐mediated signalling. We discuss experiments to test this hypothesis and potential relevance in health and disease.

## Introduction/Background

Exosomes are small (50–150 nm) membranous vesicles of endocytic origin containing or displaying a ‘cargo’ of nucleic acids and proteins that are released by cells into extracellular fluids. Exosomes are found in blood, aqueous humour, CSF, urine, amniotic fluid, breast milk, seminal fluid, saliva and malignant effusions 1. Exosomes extracted from conditioned cell culture media have been used in exosome research to study their biology. Exosomes can be characterized according to their physical properties, namely size, surface charge and density, or biologically, according to their cargo and membrane‐associated antigens. Several techniques are used to measure particle size, shape and density in a suspension of vesicles, including electron microscopy (EM), dynamic light scattering (DLS), tunable resistive pulse sensing (TRPS) and nanoparticle tracking analysis (NTA) 2. As a colloidal nanoparticle, the surface charge of the exosome is reflected by its zeta potential, which is, in turn, considered to be a characteristic property of the population of exosomes. The zeta potential of disperse systems (e.g. emulsions, suspensions and colloidal dispersions) is a measure of charge stability and affects all particle–particle interactions 3. Exosomes, like all materials, spontaneously acquire surface electrical charge when brought into contact with a polar medium, such as a hydrophilic buffer. Like the plasma membrane of cells, the exosome surface will generally be negatively charged in such buffers. The charging mechanisms include electron affinity differences of the two phases, ionization of exosome membrane surface groups and differential ion adsorption from electrolytes solutions.

In terms of scale, a higher zeta potential results in greater electrostatic repulsions between particles and minimizes their tendency for aggregation and/or flocculation. Vesicles with zeta potentials that vary between −30 mV and +30 mV tend to aggregate, although the precise threshold of stability varies according to particle type. With respect to surface charge, the stability and ability of exosomes to properly deliver signals depend on the zeta potential of the exosome, on the pH and on the ionic strength of the surrounding biological fluid 3. Large differences in size and zeta potential values have been reported for characterized exosomes derived from different body fluids 4, 5, 6, 7, tissues 8, 9 or cell cultures 10.

Cellular communication by secreted soluble proteins and membrane surface receptors is essential for physiology of multicellular organisms. Such interactions mediate a variety of processes including angiogenesis, tumour invasion and tissue proliferation. In recent years, exosomes have been shown to participate in such cellular signalling processes 11. In addition, the mechanisms by which exosomes communicate with target cells have been proposed. Exosomes can fuse with the plasma membrane of target cells, leading to the release of exosomal contents into the target cell 12. Exosomes can also serve as a transporter for aqueous insoluble ligands. For example, Wnt proteins are very lipophilic and require a lipid environment for transport. Extracellular vesicles provide an ideal vehicle to both display and transport Wnts proteins 13, 14. Other communication mechanisms whereby exosomal membrane proteins interact with the target cells include endocytosis or macropinocytosis 15. Finally, exosomal membrane proteins can be cleaved by proteases of the target cell, with the resulting fragment acting as ligands for cell surface receptors on the target cell 11. Communication specificity is mainly achieved *via* ligand–receptor interactions between vesicles and target cells. Exosome nanovesicles essentially exist as natural colloidal suspensions in most media (buffer, culture media, serum and plasma). Ensuring homogeneous dispersal of nanovesicles or nanoparticles is necessary for preserving functionality *in vivo* and for enhancing stability during storage 16. The specific interactions between exosomes we propose must be distinguished from aggregation of exosomes that occurs during extraction, storage or upon freeze and thaw 17. Indeed, cluster induction of exosomes by the elution buffer 18 and plasma proteins has been reported 19. The degree of clustering exosomes along the extraction and storage process can be minimized by proper centrifugation, filtration and final suspension in a suitable concentration 20, 21, 22.

Until recently, the gold standard for characterizing exosomes appearance and shape was transmission electron microscopy of preparations. Although dedicated techniques for extracellular vesicles size measurements are available, electron microscopy remained the method for particle form evaluation. In many instances, examples of exosome:exosome pairs can be observed 23, 24, 25, 26, 27, 28, yet these interactions were not highlighted or discussed 29, 30, 31, 32. These overlooked findings serve as a foundation for our hypothesis.

### Objective

To present a case for a mechanism by which exosomes physically interact to regulate signalling in biological systems.

### Hypothesis

Target cells release exosomes to physically interact with incoming exosomes, forming dimers that attenuate incoming exosome‐mediated signalling.

### Evaluation of the hypothesis and primary results

**Figure 1:**
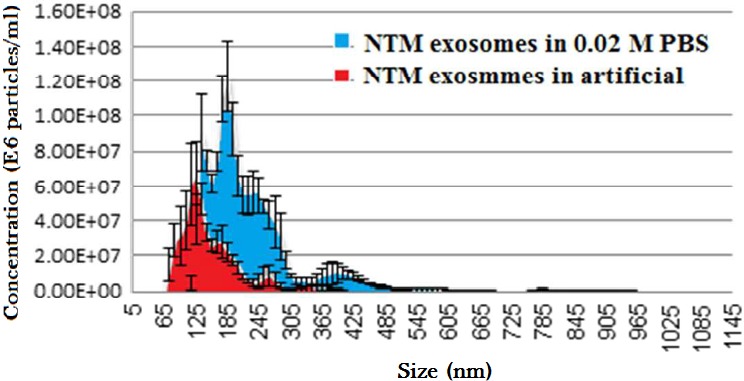
Effect of the ionic strength of two different vehicles, on the size of exosomes. Normal Trabecular Meshwork (NTM)‐derived exosome size distribution depends on the vehicle, as it was different for exosomes suspended in artificial aqueous humour (AAH; pH 7.2, IS = 0.153) and for exosomes suspended in 0.02 M phosphate buffer (PBS; pH 7.2, IS = 0.025) The size of NTM‐derived exosomes was measured with NanoSight5000 for exosomes (at the same concentration) suspended in PBS or artificial AH. An increase in exosome size was detected when the exosomes were suspended in the PBS relative to the same exosomes suspended in the artificial AH.

Several experimental steps could be taken to test our hypothesis. In our laboratory, exosome‐mediated signalling in the ocular drainage system is under investigation regarding the pathophysiology of ocular hypertension that leads to open angle glaucoma. Intraocular pressure is tightly regulated by a specialized circulatory system. Aqueous humour is produced by the ciliary epithelium and flows into the posterior chamber, which then moves into the anterior chamber and is finally drained through the trabecular meshwork (TM) and into Schlemm's canal. As a first step, conditions that favour exosome:exosome interactions need to be defined. Two approaches can be used. In one, a single population of exosomes is maintained in versions of buffer composed of different ionic strengths. For example, the phosphate‐buffered saline (PBS) buffer (NaCl 138 mM, KCl 2.7 mM, NaH_2_PO_4_ 1.9 mM, Na_2_HPO_4_ 8.1 mM) that is typically used for exosome storage has an ionic strength of 162.7 mM. The use of the same PBS buffer (pH 7.2) at 0.1 and 0.01 M concentrations, possessing ionic strengths of 16.27 and 1.627 mM, respectively, would allow for exploring the effects of ionic strength on exosome:exosome pair formation using any nanoparticle size and zeta potential assessment method, such as nanoparticle tracking analysis or tunable resistive pulse sensing. Table 1 summarizes the ionic strength effects on zeta potential values of normal trabecular meshwork (NTM) cell‐extracted exosomes. A reduction in the NTM cell exosome Zeta potential was due to the reduction in the vehicle ionic strength. This suggests that lower repulsion forces between the vesicles might be translated to higher exosomes couple and cluster formations (Table 1). Increasing the ionic strength of the exosomes buffer (0.153 for artificial aqueous humour and 0.6 for PBS 0.02 M at the same pH‐7.2), resulted with lower exosome size with higher ionic strength and higher zeta potential (Fig. 1).

**Table 1:** Zeta potentials of NTM‐derived exosomes suspended in vehicles with different ionic strengths. A reduction in the Normal Trabecular Meshwork (NTM) exosomes Zeta potential due to the reduction in the vehicle ionic strength suggests lower repulsion forces between the vesicles that might be translated to higher exosomes couple and cluster formations

	Phosphate buffer 0.1 M	Phosphate buffer 0.02 M	Artificial aqueous humour
Ionic strength	0.06	0.025	0.153
NTM exosomes Zeta potential (mV)	−24.90 ± 1.21	−21.667 ± 3.62	−28.2 ± 4.29

Phosphate buffer or artificial aqueous humour with the same pH (7.2) but with different ionic strengths was used to measure the exosomes surface charge (Zeta potential) measured with a Zetasizer ZS in two independent triplicates.

**Figure 2:**
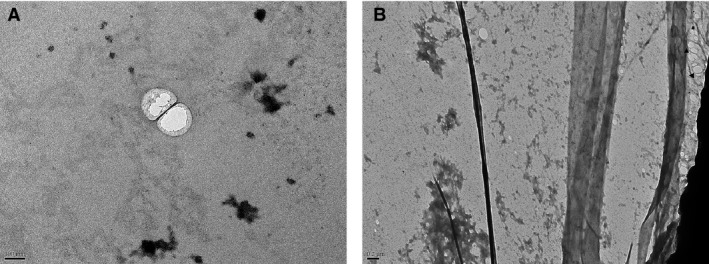
Representative physical exosome:exosome interaction by TEM analysis. Typically, exosomes isolated from conditioned media of cells appear as round vesicles of heterogeneous sizes, ranging from 30 to 150 nm. Physical exosome:exosome interactions lead to changes in exosome shape. (**A**) Non‐Pigmented Cilliary Epithelium (NPCE): Upon interaction, the round single exosomes become more flattened, with relatively a contact area between the interacting exosomes. (**B**) TM‐123: On the right side, three flattened exosome:exosome interactions can be observed (▲); non‐interacting exosomes are presented (*). Non‐pigmented ciliary epithelium cell and TM‐123‐derived exosomes were extracted by ultracentrifugation, suspended in 0.1 M PBS, pH 7.2. Picture **A** and **B** was generated by TEM.

Using a range of buffers presenting physiologically relevant pH values and exosomes for any native source, the effects on exosome:exosome formation of pH at a fixed ionic strength could be similarly evaluated. In addition, the combined importance of ionic strength and pH for exosome:exosome formation could be considered. Moreover, exosome concentration is a dominant factor in any solution tendency to form exosome:exosome complexes. Transmission electron microscopy (TEM) can be used for qualitative analysis of physical exosome:exosome interactions. Physical exosome:exosome interaction (‘mitosis like’ phenotype) can be detected using TEM by decreasing zeta potential; pH and exosome concentrations (Fig. 2).

**Figure 3:**
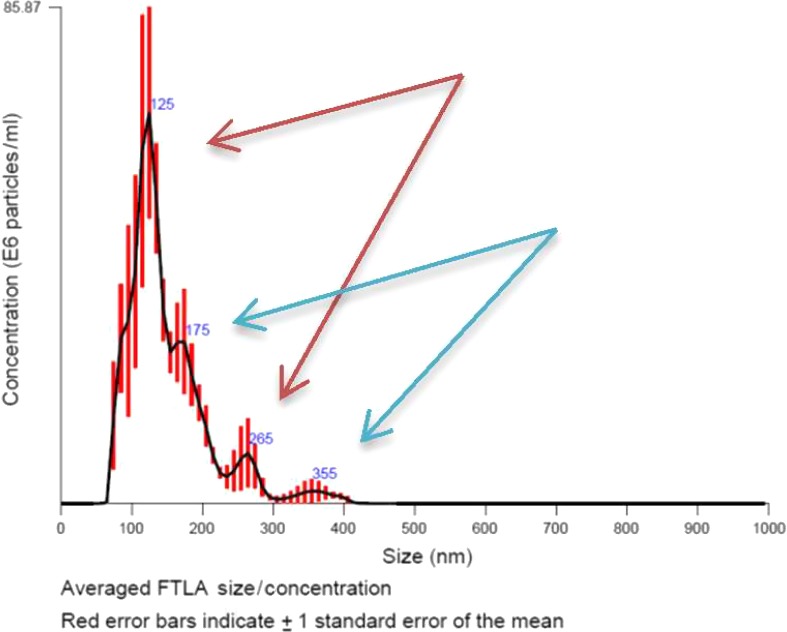
Representative graph of nanoparticle tracking analysis (NTA) analysis of Trabecular Meshwork (TM) exosomes. Normal trabecular meshwork cell‐derived exosomes were extracted. The arrow indicates possible couple forming exosomes following extraction, storing and dilution for size analysis. The amount of exosomes couple is estimated <5%.

Normal trabecular meshwork cell exosomes demonstrated possible exosome:exosome dimers using general extraction and storage 33 (Fig. 3). The optimal concentration of exosomes in solution that will favour exosome:exosome complex formation but will not dominate cluster forming during extraction and storage needs to be evaluated.

**Figure 4:**
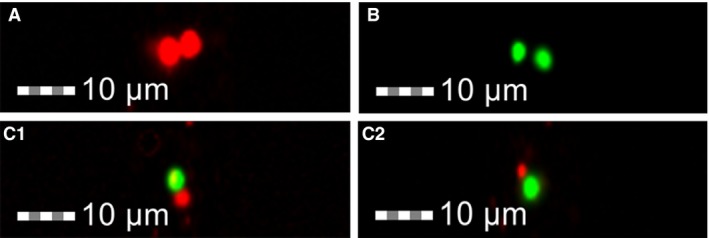
ImageStream analysis of physical exosome:exosome interactions. (**A**) DID (red)‐stained NPCE‐derived exosome and DIO (green)‐stained NTM‐derived exosome were mixed *in vitro* in a 1:1 number ratio in PBS pH = 7.2, ionic strength = 0.06 at room temperature and analysed with ImageStream. (**A**) Physical exosome:exosome interaction of DID‐stained NPCE‐derived exosomes (**B**) DIO‐stained TM‐derived exosomes demonstrate proximity without physical exosome:exosome interaction(**C**) Physical exosome:exosome interaction between NPCE‐ and TM‐derived exosomes(**C**1&**C**2).

A second set of experiments involve two separate populations of exosomes isolated from two relevant cell types or tissues that communicate, stained with different lipophilic dyes or labelled with two different antibodies. These populations could be traced by Flow Cytometry, combining functional insights into microscopy (ImageStream^®X^) for physical exosome:exosome interactions. For instance, exosomes from each source could be distinctly stained with the lipophilic membrane tracers DiD and DiO, with characteristic excitation/emission values (644/663 and 484/501 nm, respectively) to allow clear identification of the source of a given exosome. Using this approach, non‐pigmented ciliary epithelial (NPCE) or NTM cell‐derived exosomes were stained and analysed separately (Fig. 4A and B) or following of NPCE and NTM exosomes mixture at a known ratio (Fig. 4C).

Alternatively, each exosome population could be labelled by antibodies directed to exosomal protein markers, such as CD‐63, CETP, ANXA5‐1, TSG101, EpCam, Vimentin, ALIX or FLOTILLIN‐1. Another approach that probably will less impact the surface properties is by RNA labelling. The exosome‐producing cells can be cotransfected with FAM or Cy5 nucleotide‐labelled relevant miRs 26.

Finally, our hypothesis could be assessed using an *in vitro* cell model in which an exosome‐mediated effect can be easily detected and measured. Recently, we described that exosomes released by a transformed non‐pigmented ciliary epithelial (NPCE) cell line attenuates Wnt signalling in trabecular meshwork cell line, *in vitro* 34. Using this assay, the ability of TM‐derived exosomes to attenuate the Wnt signal delivered by the NPCE exosomes can be tested by artificially increasing TM‐derived exosome concentration in the TM cell‐conditioned media. Higher TM‐derived exosome concentration will increase the possibility of physical exosome:exosome interactions yielding exosome heterodimers. Riches A. *et al* reported the existence of a feedback regulatory mechanism for controlling exosome release, regulated by the presence of exosomes in the extracellular environment derived from their own cells 35. Increasing TM‐derived exosome concentration in TM culture media using the appropriate conditions of ionic strength, pH and then treatment with NPCE exosome at different ratio between TM‐ and NPCE‐derived exosomes will allow monitoring of Wnt signalling changes in TM cells and in parallel physical exosome:exosome interactions. We expect that the TM‐derived exosomes will attenuate the Wnt inhibition found when only the NPCE‐derived exosome is presented.

## Material and methods

### Cell culture

The research results are based on exosomes extracted from conditioned media of two ocular cells lines: non‐pigmented ciliary epithelium (NPCE) kindly donated by M. Coca‐Prados, Yale University, USA 36; normal trabecular meshwork (NTM) kindly donated by A.F. Clark, Alcon Research, Ltd, Fort Worth, TX, USA 37. TM‐123, human trabecular meshwork cells were isolated from donor eyes using a blunt dissection technique followed by an extracellular matrix digestion protocol 38. Cells were grown in DMEM with 10% foetal bovine serum exosome‐depleted serum containing 50 μg/ml gentamycin at 37°C in 5% CO_2_. Cells were split every 6–7 days and were studied after reaching minimal 80% confluence.

#### Exosome‐depleted serum

Media were supplemented with all nutrients and with 20% (v/v) FBS, centrifuged overnight at 100,000× *g* and 4°C, and then, the supernatant was sterilized by passing it through a 0.22 μm filter.

#### Exosome isolation

Exosome extraction was carried out using a series of ultracentrifugation steps as described previously 39.

#### Exosomes size and zeta potential determination

Nanoparticle tracking analysis (NTA) was performed with a NanoSight NS500 instrument and the NTA 2.0 analytical software. Zeta potential measurements were performed using Nano ZS (Malvern, PA, USA)**.** All experiments were performed at 1:1000 dilutions, yielding particle concentrations of ≈6 × 10^7^/ml.

### ImageStream analysis

To assess the interactions between exosomes derived from different cell types; NPCE and NTM, a mixture of labelled EV's was analysed using an ImageStream device.

Exosomes from NPCE cells‐containing pellets were suspended in 1 ml of PBS containing 7.5 μl DiD (1,10 ‐dioctadecyl‐3,3,30,30 ‐tetramethyl‐indodi‐ carbocyanine,4‐chloro‐benzenesulfonate salt Biotium, Hayward, CA, USA), while extracellular vesicles from NTM cells were suspended in the same amount of PBS containing 7.5 μl DiO (3,3′ ‐dioctadecyloxacarbocyanine, perchlorate). Exosomes were incubated with the lipophilic dyes for 10 min. in room temperature. Next, exosome‐containing pellets were centrifuged for 70 min. at 100,000 g and 4°C to remove unincorporated DiD or DiO. Labelled exosomes were re‐suspended in 300 μl of PBS. Exosome fluorescence was captured and photographed using an ImageStreamX high‐resolution imaging flow cytometer (Amnis, Co., Seattle, WA, USA). A total of 10,000 particles for each measurement were excited using 642 nm laser beam for exosomes membrane labelled with DiD and 488 nm laser beam for exosomes membrane labelled with DiO.

### Transmission electron microscopy

For transmission electron microscopy, freshly isolated exosome suspensions were fixed in 4% paraformaldehyde for 1 hr. Exosome suspensions (approximately 5 μl) were applied to copper mesh Formvar‐coated carbon‐stabilized grids, were allowed to adsorb to the grid for 4–5 min. and then were wicked off with filter paper. For negative staining of exosomes, 1% aqueous uranyl acetate (5 μl) was applied to the grid for 30 sec. and then wicked off with Whatman filter paper. Grids were allowed to thoroughly dry before viewing.

## Discussion

We hypothesize that physical exosome:exosome interactions regulate the actions of exosomes in signal transfer. This hypothesis is beyond the currently accepted biological role for exosomes in cell–cell communication. Physical interaction between vesicles, in general, and exosomes, in particular, is a well‐described phenomenon. The novelty of our hypothesis is that exosome:exosome interactions occur in a selective manner, meaning that the target cell controls the incoming signals mediated by exosomes through the release of its own population of exosomes, which physically intercept the incoming signalling exosomes. In this scenario, the exosome:exosome interaction is based on the particle size, zeta potential and/or ligand‐receptor pairs. We hypothesize that physical interactions between exosomes will have an inhibitory effect on incoming signals, due to the increase in particle size that will mask exosome‐binding ligands and make fusion, endocytosis or micro‐pinocytosis more difficult. In other cases, exosome:exosome complexes might expose specific ligand to protease cleavage and enhance exosome‐mediated signalling. Whether the particle pairs fuse to create a large particles is another possibility that needs further research. Exosome release by cells is a general process that normally contributes to cellular communication. Our proposed mechanism tempers the maximal potential of signals delivered by exosomes from distant cells due to these physical exosome:exosome interactions as part of general tissue homoeostasis and possibly dysregulated during pathological conditions such as cancer.

## Conclusions

In conclusion, our hypothesis suggests that exosome:exosome interactions act as exosome‐mediated signalling modulator. Such signalling modulation might have significant influence on exosome‐mediated biological processes such as cancer metastasis, cancer immunoregulation, intraocular pressure homoeostasis, tissue regeneration and many others.

## Conflicts of interests

The authors confirm that there are no conflicts of interest.
